# Unraveling the Resistome, Virulome, and Pathogenicity of *Escherichia Coli* O157:H7 From Cattle Feces

**DOI:** 10.1155/ijm/5087461

**Published:** 2025-02-05

**Authors:** Bukola Opeyemi Oluwarinde, Daniel Jesuwenu Ajose, Tesleem Olatunde Abolarinwa, Peter Kotsoana Montso, Henry Akum Njom, Collins Njie Ateba

**Affiliations:** ^1^Food Security and Safety Focus Area, Faculty of Natural and Agricultural Sciences, North-West University, Mahikeng, South Africa; ^2^Department of Microbiology, Antimicrobial Resistance and Phage Bio-Control Research Group (AREPHABREG), North-West University, Mahikeng, South Africa; ^3^Agricultural Research Council, Private Bag X1251, Potchefstroom, South Africa; ^4^School of Biology and Environmental Sciences, Faculty of Agricultural and Natural Sciences, University of Mpumalanga, Mpumalanga, South Africa

**Keywords:** antimicrobial resistance, *Escherichia coli* O157:H7, foodborne diseases, pathogenicity, Shiga toxins, whole genome sequencing (WGS)

## Abstract

Antimicrobial-resistant *Escherichia coli*, especially those belonging to the serotype O157, are increasingly linked to foodborne diseases with significant fatality rates worldwide. The food and medical industries have focused on *E. coli* O157:H7 due to its ability to produce toxins coupled with its low infectious dose. The aim of this study was to assess the virulome, resistome, and pathogenicity of *E. coli* O157:H7 using whole genome sequencing. Three previously isolated *E. coli* O157:H7 strains from cattle feces were subjected to whole genome sequencing. The genome sizes of all three *E. coli* O157:H7 strains were 5,117,276 bp, 5,039,443 bp, and 5,034,351 bp. The C + G contents were 50.22%, 50.53%, and 50.54%, while the number of contigs was 110, 43, and 42, respectively, for *E. coli* O157:H7 strains J32, J57, and J69. Several virulence determinants (hemorrhagic *E. coli* pilus (HCP), eaeA, hemolysin, etc.) were found in the genomes of these isolates. In addition, antibiotic resistance genes conferring resistance to aminoglycosides, tetracyclines, macrolides, fluoroquinolones, penams, carbapenems, cephalosporins, cephamycin, rifamycin, phenicols, monobactams, and nitroimidazole were found in the genomes. Interestingly, the genomes of these isolates also harbored determinants encoding resistance to disinfectants and antiseptics, indicating their concern in the food production and medical sectors. This highlights the public health concerns of these isolates, indicating the need for constant surveillance.

## 1. Introduction

Foodborne diseases resulting from the consumption of contaminated food are currently a growing public health concern, posing considerable socioeconomic impacts through strains on healthcare systems, lost productivity, and negative effects on tourism and trade, with over 200 different types of complications reported yearly [1]. Among foodborne pathogens, *Escherichia coli* especially those belonging to the serotype O157:H7 have been identified as one of the most significant causes of these complications accounting for severe illnesses and high mortality rates among humans [2]. The frequent isolation of this emerging pathogen among clinical samples and the food chain demonstrates high adaptability and genetic variability, leading to its ever-increasing prevalence worldwide [3]. The public health significance of these pathogens is amplified by the challenges they pose in both developing and developed countries, despite advanced public health policies in the latter [4, 5].

Cattle are known to be the main reservoir for *E. coli* O157:H7, with fecal excretion of this pathogen reported to be transient, contributing to its significant public health impact [6]. In addition, the recent increase in antimicrobial resistance (AMR), especially in intensive dairy farming, is largely due to the misuse of antibiotics aimed at treating *E. coli* O157:H7 infections. This issue is exacerbated by the increasing demand for animal protein, especially in developing countries, necessitating adherence to strategies designed to combat AMR and promote “good health and well-being” [6, 7]. Transmission of *E. coli* O157:H7 to humans often occurs through the consumption of contaminated food and water, person-to-person contact, and direct or indirect contact with animals [8]. The presence of this serotype in pets also poses a risk to humans [8]. Shiga toxin–producing *E. coli* (STEC) has been identified as the ninth most prevalent cause of foodborne illnesses, the fourth most common cause of hospitalization, and the third most significant cause of fatalities [9]. Despite significant attention on enterohemorrhagic *E. coli* (EHEC), specifically *E. coli* O157:H7 globally, recent studies have demonstrated that other non-O157:H7 strains including O26, O45, O91, O103, O104, O111, O113, O121, O118, O128, O145, O148, and O174 have acquired genetic traits that make them pathogenic to humans [10, 11]. These strains harbor a variety of virulence determinants including stx_1_, stx_2_, hlyA, and eaeA that generally enhance their capacity to produce infections such as diarrhea, hemorrhagic *coli*tis (HC), and hemolytic uremic syndrome (HUS) in humans [12, 13].

Putative pathogenic determinants associated with *E. coli* O157:H7, such as intimin encoded by eaeA, a plasmid-encoded enterohemolysin (hlyA), and a pore-forming cytolysin encoded by the clyA gene, play significant roles in the pathogenicity of this strain [14]. While the hlyA gene is responsible for the disruption of eukaryotic red blood cells and the inhibition of protein synthesis, eaeA facilitates the adhesion and invasion of intestinal epithelial cells by the bacterium, resulting in severe detrimental consequences [15]. Given the frequent misuse of antibiotics, both in humans and the agriculture sectors even in Africa, [16], calls to combat antibiotic resistance may struggle to yield valuable outcomes [17]. However, surveillance of *E. coli* O157:H7 [18] and joint efforts to enhance the search for new and alternative nonantibiotic agents are critical in the fight against this increasing global public health threat [19]. Hence, knowledge of the genetic variability of *E. coli* O157:H7 and their resistance and virulence profiles that contribute to the pathogenesis of these organisms may be of great epidemiological importance.

Conventional molecular methods, such as polymerase chain reaction (PCR) and 16S rRNA gene sequencing have been useful in the detection and characterization of the resistance and virulence profiles of *E. coli* O157:H7 [20]. However, whole genome sequencing (WGS) has revolutionized studies on AMR genes, virulence, and other pathogenicity determinants, as well as investigations aimed at evaluating the genetic variability of an organism's complete genome, including *E. coli* O157:H7 [21]. WGS has significantly contributed to public health response systems, mainly due to its robustness in determining point source contamination during infection control, and therefore is very valuable in realizing of the “One Health concept” [22]. Extensive data have been generated on the pathogenicity of environmental and clinical Shiga toxin–producing and biofilm-forming *E. coli* especially those belonging to the serotype O157 [20] and O177 [23], using targeted PCR assays. This study aims to expand on previous investigations by using next generation sequencing (NGS) techniques to assess the genome of *E. coli* O157:H7 and characterize the virulome, resistome, and pathogenicity of the genomes. The study focused on profiling key virulence genes, including *eaeA, fimH*, *hlyE,* and *clyA,* critical for attachment, host colonization, and toxin production in the genome of these isolates. Analysis of the resistome was designed to determine the presence of antibiotic resistance genes, especially those conferring resistance to beta-lactams, tetracyclines, and aminoglycosides. Furthermore, the aim was to assess the association of virulence and resistance on the overall pathogenicity of *E. coli* O157 and its potential to cause severe disease outcomes.

## 2. Materials and Methods

### 2.1. Ethics Statement

Ethics approval for the study was obtained from the North-West University Animal Research Ethics Committee (NWU-AnimCareREC), and the study was assigned ethics number NWU-00772-23-A5.

### 2.2. Bacteria Isolates

Three *E. coli* isolates were obtained from the culture collection of the Antimicrobial Resistance and Phage Bio-control Research Group (AREPHABREG). These isolates had been previously isolated and characterized using PCR [24].

### 2.3. Revival of *E. coli* O157:H7 Isolates

For the resuscitation of *E. coli* O157:H7 strains, glycerol stocks of the cultures were streaked onto Sorbitol–MacConkey agar (SMAC) (Merck, South Africa). The plates were incubated aerobically at 37°C for 24 h. Following incubation, the plates were carefully inspected for distinct pink colonies, demonstrating the macroscopic characteristics of the target organism. Distinct *E. coli* O157:H7 colonies were further purified by subculturing onto SMAC and incubated aerobically at 37°C for 24 h. Two pink colonies were randomly selected, inoculated into the sterile nutrient broth, and used for further studies.

#### 2.3.1. DNA Extraction and WGS of *E. coli* O157:H7

Chromosomal DNA was extracted from overnight cultures of *E. coli* at room temperature (28°C) using the ZR Genomic DNA-Tissue Miniprep Kit (Zymo Research Corp., Irvine, California, United States of America) following the manufacturer's instructions. DNA concentrations were quantified using a NanoDrop^TM^ Lite spectrophotometer (Thermo Scientific, Walton, Massachusetts, United States of America) following the manufacturer's instructions, and the purity was determined. The ratio of the optical density of DNA extracted at 260/280 was 1.8, and 260/230 was between 2.0 and 2.2, indicating good quality DNA. Purified DNA samples were transported on ice to the Agricultural Research Council's (ARC) Biotechnology Platform (ARC-BTP) at the Onderstepoort Veterinary Institute in South Africa, where sequencing was performed. The DNA samples were used to create Libraries using the MGIEasy UDB Universal DNA Library Prep Kit according to the manufacturer's instructions. Aliquots of 80 μL (500 ng) of gDNA were fragmented with the Covaris, and the fragmented gDNA was size-selected with magnetic beads. After size selection, approximately 50 ng of 280 bp DNA fragments were obtained. Paired-end sequencing was performed on the MGI DNBSEQ-G400 (312 cycles) following the manufacturer's protocol to generate data of 1905 MB, 594.53 MB, and 969.34 MB for isolates J32, J57, and J69, respectively.

#### 2.3.2. Analysis of WGS Data

Following WGS, the raw sequences were collected and uploaded to the Kbase platform (V 2.1) (https://kbase.us/) [25]. FastQ Reads (V 0.11.5) were used for quality control of the raw reads using the parameters: Phred quality score (QS30 > 75%), cluster density (600–1300), and clusters passing filters (> 80%) [26]. Trimmomatic (V 0.36) was used to filter and trim the adapters and ambiguous nucleotide sequences at default parameters (poor quality sequence: 0.05 and ambiguous nucleotides, maximum of two nucleotides included) [27]. The cleaned raw data were submitted to the National Center for Biotechnology Information (NCBI) database (https://www.ncbi.nlm.nih.gov/) to obtain sequence reads archive (SRA) accession numbers.

#### 2.3.3. Assembly and Annotation of the Genome

The SPAdes online tool (Version 3.13.0) was used to assemble the genome from the trimmed (high-quality) data with a minimum contig size threshold of 200 bp [28]. The Prokaryotic Genome Annotation Pipeline (PGAP) [29] and Rapid Annotation using Subsystem Technology (RAST V 2.0) Server [30] were employed for gene prediction and genome annotation using default parameters. The circular genome maps of the isolates were generated by uploading the annotated genome to the online database Proksee: Genome Analysis for Circular Genome Visualization (CGView) [31].

High-quality genome sequences of other *E. coli* O157:H7 isolates were selected and downloaded from the NCBI database. These sequences were included in constructing a phylogenetic tree. The phylogenetic tree was constructed using the Archaeopteryx.js online tool on PATRIC [32]. MUSCLE was used to align the sequences, fast bootstrapping was utilized to create the support values in the tree, and RaxML was used to evaluate the matrix.

#### 2.3.4. Accessing the Virulome, Resistome, and Pathogenicity of the *E. coli* O157:H7 Isolates

Online tools from the Center for Genomic Epidemiology (CGE) (https://www.genomicepidemiology.org) were used to assess the genomes of *E. coli O157:H7* isolates. The contigs were uploaded to the CGE platform, and several tools were used to access the genomes. The Mobile Element Finder V 1.0.3 [33] was used to detect mobile genetic elements (MGEs) in the genomes, while the VirulenceFinder V 2.0 [22] was used to assess virulence genes in the isolates with parameters set at a 90% threshold for %ID and a 60% minimum length. Antibiotic resistance genes were assessed using ResFinder V 2.2 [34], while the presence of plasmids in the bacterial genomes was assessed using PlasmidFinder V 2.1 [33].

PathogenFinder V 1.1 was used to estimate organism pathogenicity to human hosts [35]. The pathogenic Virulence Factor Database (VFDB) was also accessed on VFDB (mgc.ac.cn) to provide further data linked to the studied genomes' virulence. Phage Search Tool Enhanced Release (PHASTER) (https://phaster.ca/) was used to detect the presence of prophage sequences. The genomes were scanned for whole or intact, questionable, and incomplete prophage sequences and were automatically classified into intact (score > 90), questionable (score 70–90), and incomplete (score < 70) prophages, respectively, based on their sizes, similarity to known phages, and the presence of phage-like and phage cornerstone genes [36].

## 3. Results

### 3.1. WGS of *E. coli* O157:H7 Strain

Following the analysis of the sequence data from WGS procedures, information detailing the overall genomic characteristics of the isolates was obtained. This includes the presence of virulence genes, resistant genes, and toxigenic genes in the genomes. In addition, these findings revealed the presence of various plasmids and prophages in the genomes as well as genetic similarities between the *E. coli* O157:H7 in this study and other *E. coli* O157:H7 strains from previous studies.

#### 3.1.1. Genomic Characteristics of *E. coli* O157:H7 Strain

**Figure 1:**
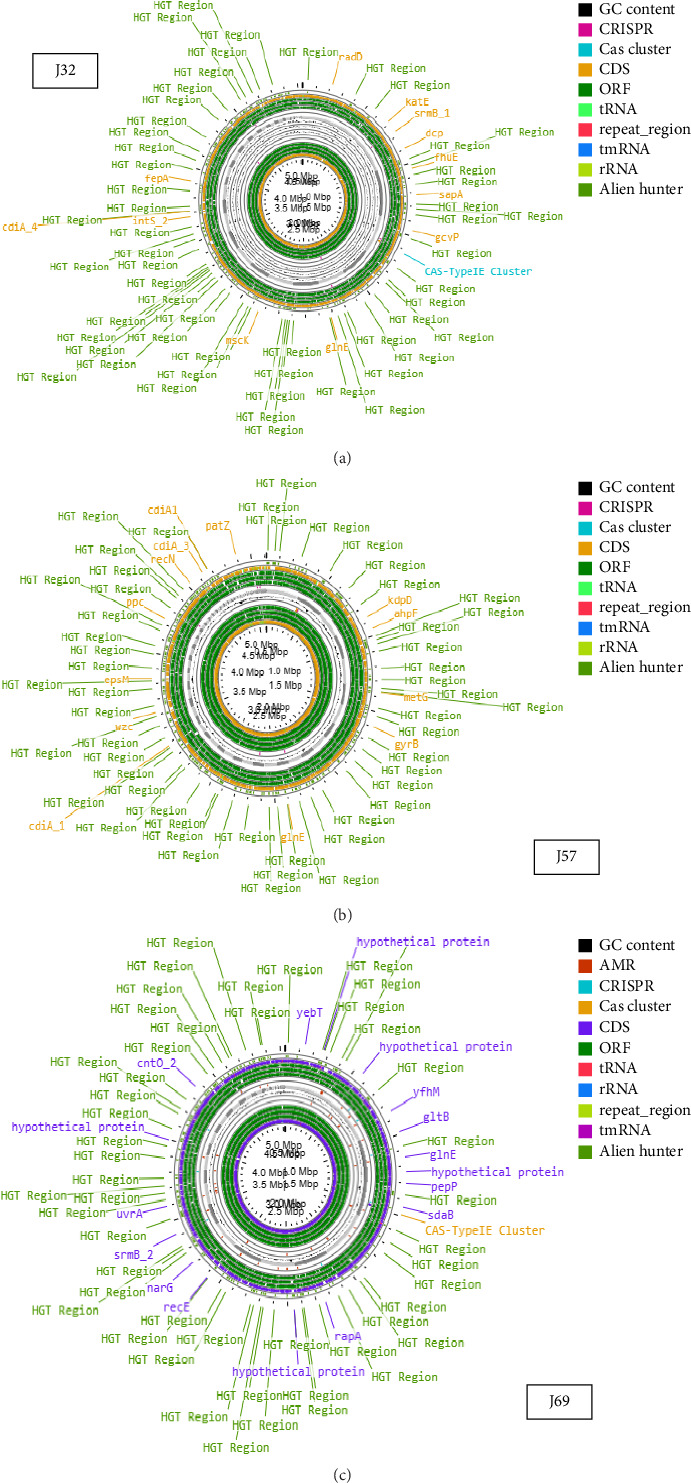
The circular genome map of *E. coli* (J32, J57, and J69) isolates. Circle displays from inside to outside: GC content (black), drug antimicrobial resistance genes (red), CRISPR (turquoise blue), cas cluster (yellow), CDS (purple), ORF (green) tRNA (red), rRNA (blue), repeat region (lemon), tmRNA (light purple).

**Figure 2:**
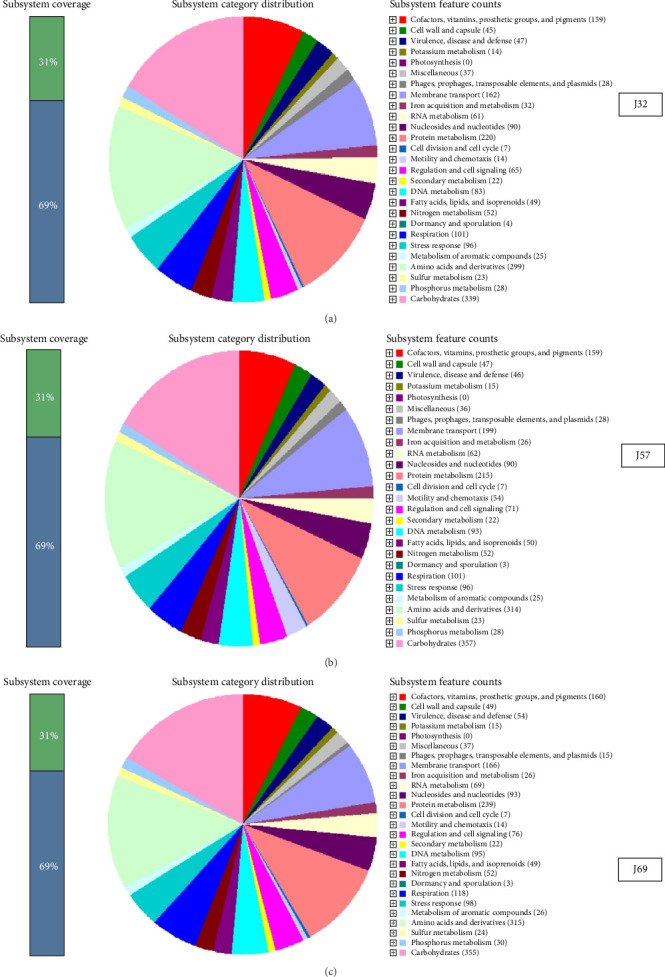
The genome characteristics of the *E. coli* O157 strains (J32, J57, and J69) associated with specific subsystems and their distribution across various categories.

The genome sizes of the *E. coli* O157:H7 strains were 5,117,276; 5,039,443; and 5,034,351 bp for J32, J57, and J69, respectively (Table 1). The GC contents for the isolates were 50.22% (J32), 50.53% (J57), and 50.54% (J69). The circular genomes of the isolates were visualized on the online CGView database [31], as shown in Figure 1. In addition, the genomic characteristics are linked to specific subsystems, and their distribution across various categories is depicted in Figure 2. The genome sequences were deposited into the NCBI GenBank database under the accession numbers JAVLRS000000000, JAVCZL000000000, and JAURAD000000000 for J32, J57, and J69, respectively.

#### 3.1.2. Virulence Factors and Associated Genes of *E. coli* O157:H7 Strain

Analysis of the bacteria genomes using the VFDB (https://www.mgc.ac.cn/VFs/), a reference database for assessing bacterial virulence factors, revealed that a total of 28 virulence factors and several genes were present in the genomes.  Table 2 presents the virulence factors and their associated genes that were detected in the genomes during the study. Generally, several determinants encoding toxin production, adherence, autotransportation, invasion, iron uptake, non-LEE–encoded TTSS effectors, and secretion systems were detected in the genomes of these isolates. The genome of isolate J69 exhibited a substantial proportion (84%) of virulence genes, whereas the genomes of isolates J32 and J57 contained 78.5% and 80% of the accessed virulence genes, respectively. All three genomes harbored *eaeA* and *fimH* genes. In addition, the genomes were also found to contain the toxin genes *hlyE* and *clyA*, which are responsible for the production of hemolysin and cytolysin, respectively.

#### 3.1.3. Antibiotic Resistance Genes Detected in *E. coli* O157:H7 Strain

**Figure 3:**
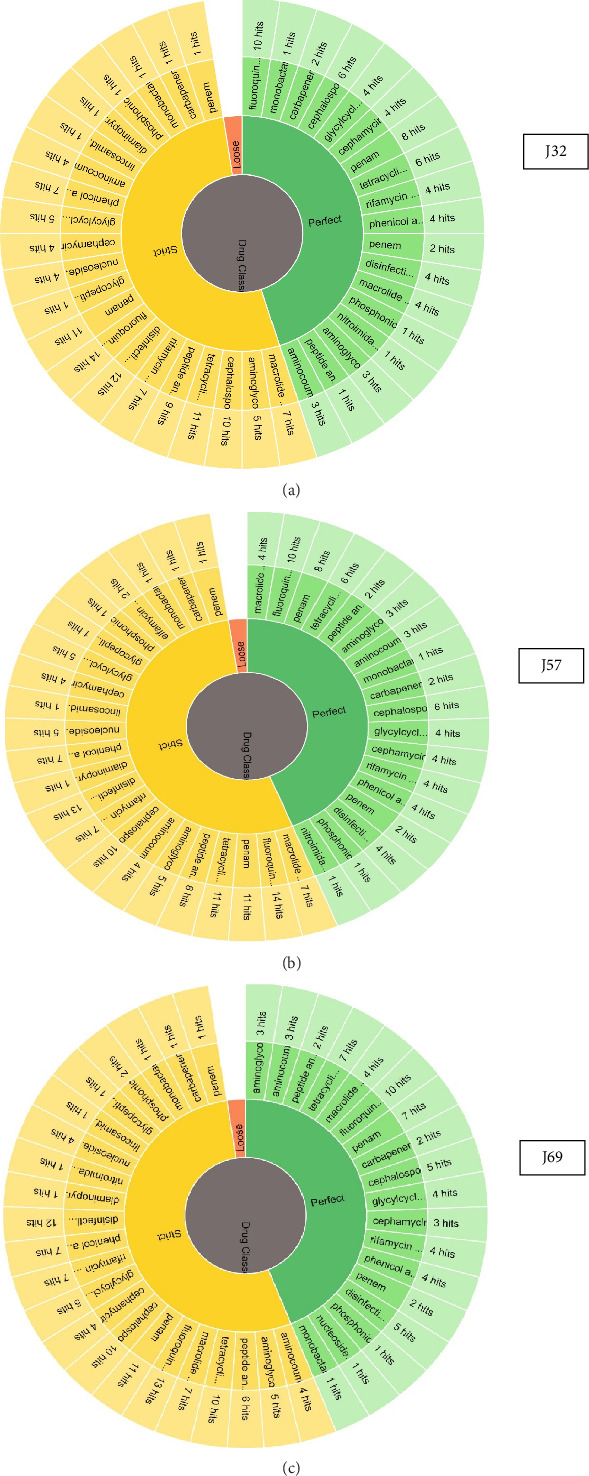
Distribution of the different classes of antibiotics in the genomes of *E. coli* O157:H7 (J32, J57, and J69).

The online Antibiotic Resistance Gene Identifier (RGI) and ResFinder analysis predicted that all three genomes carried multiple genes associated with antibiotic resistance in bacteria isolates. The three *E. coli* genomes harbored antibiotic resistance genes belonging to 17 classes of antibiotics (Table 3). The *E. coli* O157-J32 genome carried antibiotic resistance genes associated with 15 classes of antibiotics, while J57 harbored resistant genes linked to 16 antibiotic groups, and J69 harbored resistant genes belonging to all 17 classes of antibiotics. The predominant antibiotic resistance genes conferring resistance to aminoglycosides, tetracyclines, macrolides, fluoroquinolones, penams, carbapenems, cephalosporins, cephamycin, rifamycin, phenicols, monobactams, and nitroimidazole were detected in the genomes. Interestingly, the genomes of these isolates also harbored determinants encoding resistance to disinfectants and antiseptics, indicating their concern in the food production and medical sectors. Mutations in gene sequences that confer AMR were also detected in these bacterial genomes. These results provide new insights into the molecular mechanisms underlying antibiotic resistance in *E. coli* O157, which could inform future strategies for combating bacterial infections. The distribution of AMR classes that were detected in the sequenced *E. coli* O157:H7 genomes is shown in Figure 3. A list of the mutated genes that confer AMR is summarized in Table 4. The PathogenFinder identified all three isolates as human pathogens, indicating their pathogenicity and public health significance.

#### 3.1.4. Plasmids and Prophages Found in the Genomes of *E. coli* O157:H7 Strain

**Figure 4:**
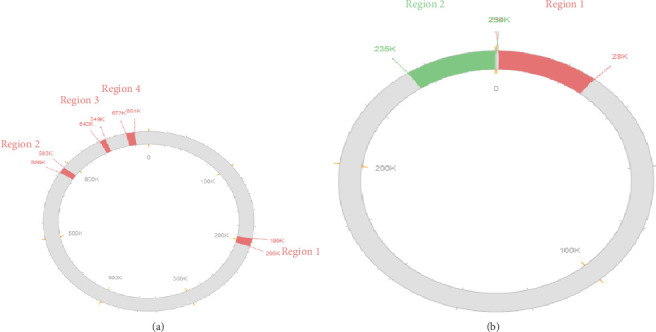
Prophage regions found in *E. coli* O157:H7 genomes.

PlasmidFinder was used to determine the presence of plasmids in each genome. The *E. coli* O157-J32 genome carried one plasmid (IncFII), while *E. coli* O157-J57 harbored four plasmids (IncFIA, IncFIB [AP001918], IncFIC [FII], and IncFII [pCoo]), and the *E. coli* O157-J69 genome possessed four plasmids (IncFIA, IncFIB [AP001918], IncFIC [FII], and IncY). The three genomes possessed 4, 5, and 12 prophage regions (J32, J57, and J69, respectively). The *E. coli* O157-J32 genome possessed three incomplete and one intact sequence, while *E. coli* O157-J57 had four incomplete and one questionable prophage sequence, and *E. coli* O157-J69 had five intact and seven incomplete sequences.  Figure 4(a) shows a diagrammatic representation of four incomplete prophage regions in a node, while Figure 4(b) shows a node with one incomplete prophage region and one intact prophage region.

#### 3.1.5. Phylogenetic Analysis of *E. coli* O157:H7 Strain

**Figure 5:**
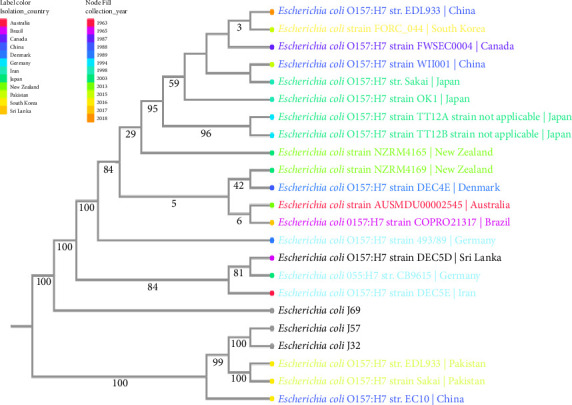
Phylogenetic tree of *E. coli* J32, J57, and J69 showing genomic similarities with *E. coli* O157 strains from other countries.

Genomic comparisons using MEGA 11 showed that *E. coli* O157:H7-J32 and J57 are similar to each other and closely related to *E. coli* O157:H7 str. EDL933 and *E. coli* O157:H7 str. Sakai, both isolated in Pakistan, as well as *E. coli* O157:H7 str. EC10, isolated in China. On the other hand, *E. coli* O157:H7-J69 was closely related to the *E. coli* O157:H7 strain DEC5E isolated in Iran (Figure 5).

## 4. Discussion

Cattle, sheep, and goats have been reported as the main reservoirs of *E. coli* O157:H7 [20]. The presence of *E. coli* O157:H7 in cattle farms has been the focus of several studies, particularly reports on the virulence and resistance genes found in *E. coli* O157:H7 [37]. The global morbidity associated with this serotype has been identified as a significant threat to human well-being [38]. In addition, the use of NGS technology, specifically WGS, has greatly improved food safety and healthcare by accurately identifying and classifying foodborne pathogens [39]. Therefore, WGS has the potential to enhance our understanding of the genomic composition of foodborne pathogens [40]. Several studies have utilized WGS to annotate unidentified genes and predict their functions through various in silico bioinformatic techniques [41, 42].

In this study, the analysis of WGS was conducted to determine the complete DNA sequence of three *E. coli* O157:H7 isolates (J32, J57, and J69) using Illumina's MiSeq platform. The genome sizes were 5,117,276 bp, 5,039,443 bp, and 5,034,351 bp with 110, 43, and 42 contigs for the J32, J57, and J69 genomes, respectively. When compared with previous studies [43], the genome sizes observed in this study were lower, which may be due to the genomic diversity among *E. coli* strains, especially when pathogenicity islands or MGEs are absent. Furthermore, genome reduction may occur as a result of nonessential gene loss as an adaptive strategy to a specific host environment. The GC content for each genome was 50.22% (J32 genome), 50.53% (J57 genome) and 50.54% (J69 genome). Understanding the variation in genome size in pathogenic *E. coli* could have significant implications for developing rapid diagnostic tools and targeted antimicrobial treatments. In terms of pathogenicity, the PathogenFinder identified all three isolates as human pathogens. *E. coli* O157:H7 (J69) had a 93.6% probability of being a pathogen, J57 had a 93.9% pathogen probability, and J32 had a 94.4% pathogen probability. This further confirms that *E. coli* O157:H7 is highly pathogenic and a public health concern.

*E. coli* strains possess virulence factors that are responsible for various processes such as colonization, adherence, invasion, persistence, and toxin production within the host [44]. The chromosome of the three *E. coli* genomes characterized, carries different virulence genes that have been detected in EHEC *E. coli* and other Diarrheagenic *E. coli*, including the long polar fimbriae (*lpfA*) gene which mediates initial contact and attachment between the pathogen and the host cell [45]. They also possess *coli* surface antigens such as Type 1 fimbriae (*fimH*) and *E. coli* common pili (*ecpA*), which promote the initial attachment, adhesin, and colonization of ETEC to the host intestinal epithelial cells [46]. The presence of the outer membrane protein (*ompT*) gene, which encodes an aspartyl protease located on the outer membrane of *E. coli*, was observed in all three genomes of *E. coli*. Omptins, by virtue of their extracellularly oriented active sites, play a significant role in promoting virulence through the enzymatic cleavage of diverse proteins, peptides, and capsular genes [47]. Interestingly, *E. coli* O157 (J69) possesses the virulence gene regulator (*eilA*) and the *air* gene that encodes enteroaggregative immunoglobulin repeat protein in EAEC and other pathogenic *E. coli* strains [48]. Other virulence factors present and used by pathogenic *E. coli* to colonize and cause disease in animals and humans are the intimin (enterocyte attaching and effacing protein [*eaeA*]) and hemolysin toxins. The possession of virulence genes confers on an organism the capacity to cause infection in humans [49]. Although the specific mechanisms by which this pathogen causes disease are not yet understood, the presence of several virulence genes/toxins in *E. coli* O157:H7 strains has been shown to significantly contribute to the pathogenicity of this bacterium and has also been linked to human illness outbreaks [50]. Our findings identify key virulence genes such as hemolysin toxin and adhesins, which are crucial for the bacteria's ability to cause severe gastrointestinal disease. The findings further provided insights into the pathogenicity and virulence mechanisms of *E. coli* O157. However, further research is needed to explore the functional roles of newly identified virulence factors.

The use of antibiotics serves the purpose of therapeutic intervention for infectious ailments in both human and animal populations; however, the no-therapeutic utilization of antibiotics has led to a rapid increase in antibiotic resistance [51]. In light of this, WGS showed the presence of several resistant genes and mutations in genes that confer AMR in the genomes studied. The AMR gene folP was found in all genomes analyzed in this study. The presence of the folP gene is associated with resistance to sulfonamides, which are synthetic chemotherapeutic agents that work as competitive inhibitors of the dihydropteroate synthase (DHPS) enzyme. The gyrA, ParC, and ParE proteins are necessary for proper chromosome partitioning in bacteria [52]. While the gyrA and ParE genes were found in the DNA sequences of all *E. coli* samples without mutations, mutations were found in the ParC genes. Studies have shown that the mutations in the gyrA and ParC genes play an essential role in resistance to ciprofloxacin [53]. Tchesnokova et al. [54] and Imkamp, Bodendoerfer, and Mancini [55] have also shown the presence of ParC genes with mutations in *E. coli* isolates from food products, humans, and animals. The presence of this mutation in *E. coli* from all three sources shows a global health issue. In addition, mutations were found in the 16S_rrsH gene in all three *E. coli* genomes. Mutations in the rrsH gene suppress streptomycin dependence and cause increased translational error frequencies in bacteria [56]. The several AMR genes found in the genomes including those encoding for *β*-lactamases and efflux pumps demonstrate that the *E. coli* O157:H7 strains in this study are multidrug-resistant. While this can facilitate the transmission of resistance genes across animals, humans, and the environment [57], this could potentially enhance the virulence of these pathogens and pose a greater burden on human health. Specifically, it heightens the risk of infection in individuals with compromised immune systems and reduces the available treatment options for associated illnesses. This enhances our understanding of the mechanisms through which the pathogen acquires and spreads resistance. It also highlights the need for enhanced surveillance of *E. coli* O157 strains, particularly those exhibiting multidrug resistance, to better manage future outbreaks. The identification of key resistance genes necessitates innovative strategies to combat AMR, thereby improving clinical outcomes for infected patients [58]. Further research is, however, needed to understand how resistance genes interact with other genomic elements to drive AMR in various environments.

In addition to genomic DNA, plasmids are present within living organisms, and they facilitate the transfer of genetic material between different organisms [59]. The presence of plasmids facilitates the proliferation of antibiotic resistance, virulence factors, and other traits within bacterial populations [60]. Moreover, the pathogenicity of bacteria is increased by the presence of plasmids that contain genetic material responsible for encoding virulence factors [61]. In this study, the analyzed genomes of *E. coli* O157:H7 revealed variations in both the quantity and makeup of plasmids. Plasmids harboring virulence genes were identified within the genomes of all three organisms. The bacterium *E. coli* O157:H7 strain J32 harbored the IncFII plasmid, carrying both the anr and traT virulence genes. *E. coli* O157:H7 strains J57 and J69 were found to have four plasmids, namely, IncFIC (FII), IncFII (pCoo), IncFIB (AP001918), and IncFIA, containing virulence genes such as ompT and traJ. The traT gene located on the *E. coli* J32 plasmid has been found to be associated with pathogenesis, specifically in relation to resistance, phagocytosis, and biofilm formation [62]. It is important to note that the IncF plasmid group encodes addiction systems that use toxin–antitoxin factors and has mechanisms for independent replication [63]. Based on the virulence genes found, *E. coli* O157:H7 infections may have serious consequences, especially in immunocompromised patients. These plasmids can also increase the transmission of genetic elements between the *E. coli* O157 strains and other bacterial species.

WGS provides new insights into taxonomic classification and genetic similarities of bacterial strains. Genomic comparisons showed that *E. coli* O157:H7-J32 and J57 are more closely related to *E. coli* O157:H7 str. EDL933, *E. coli* O157:H7 str. Sakai, and *E. coli* O157:H7 str. EC10. Both *E. coli* O157:H7 str. EDL933 and *E. coli* O157:H7 str. Sakai isolates were detected in Pakistan, while *E. coli* O157:H7 str. EC10 was isolated in China. On the other hand, *E. coli* O157:H7-J69 was closely related to the *E. coli* O157:H7 strain DEC5E isolated in Iran. This finding affirms the potential migration of AMR strains across borders. Singh [64]. Recently, there has been a significant influx of Pakistanis into South Africa, driven by perceived business opportunities and political stability. In addition, there has been a rise in trade between South Africa and China, further strengthening the ties between the two countries [65]. This interconnectedness between nations could facilitate the exchange of genetic materials and potential migration of pathogens, possibly explaining the similarities between the pathogens in this study and those in other countries. However, this evidence alone is not sufficient to confirm the cross-border transmission of AMR strains, highlighting the need for further investigation. Studying transmission dynamics, especially in regions with high levels of human migration, could help elucidate the role of global movement in the spread of AMR.

## 5. Conclusion

This study provided evidence that *E. coli* O157:H7 strains obtained from bovine feces possess virulence and AMR genes. WGS of *E. coli* O157:H7 isolates provided detailed insights into the pathogenicity of these strains. The analysis indicated that the *E. coli* O157:H7 strains were classified as human pathogens assigned to the category of EHEC. Furthermore, the genome of the *E. coli* O157:H7 isolates exhibited a multitude of virulence genes (hemolysins, adhesins etc.) and AMR genes (*TolC, baeS, cpxA* etc.), as well as diverse plasmid types and prophage sequences. The observed resistance to antibiotics, which are frequently prescribed for the treatment of *E. coli* O157:H7 infections in adults, poses a significant public health issue. The public health implications of these findings are significant, particularly in the areas of surveillance, treatment, and prevention of *E. coli* O157 infections. By identifying key virulence and resistance mechanisms, this study provides critical insights that could inform the development of more effective diagnostic tools, treatment guidelines, and food safety protocols.

Furthermore, the detection of AMR genes highlights the importance of antimicrobial stewardship and the need for public education to mitigate the spread of resistant strains. These findings could influence public health policy by guiding more stringent food safety regulations, monitoring systems, and international efforts to control the spread of AMR pathogens. Findings from this study indicate that *E. coli* O157:H7 present in Mafikeng, North-West, South Africa, is highly pathogenic. It is recommended that farm owners are routinely sensitized about hygienic protocols in both farms and slaughterhouses. To ensure the safety of food, surveillance of foodborne pathogens such as *E. coli* O157:H7 in food products should be encouraged. Future perspectives include the use of alternative therapies such as bacteriophage and nanoparticles to combat infectious *E. coli* O157:H7. This will help mitigate the public health issue of antimicrobial resistance.

Limitations of the study include the sample size of *E. coli* O157 isolates. While the strains analyzed provide valuable insights, a larger and more diverse collection of isolates from different regions and environments would strengthen the generalizability of the findings. In addition, the study did not include detailed analyses of transmission dynamics, which are important for understanding how *E. coli* O157 spreads across populations and borders. Future research should explore the transmission patterns of AMR strains to inform public health interventions.

## Figures and Tables

**Table 1:** Taxonomy and genomic features of three *E. coli* strains isolated from cattle feces.

Genomic feature	Sample		
	*E. coli* O157:H7 strain J32	*E. coli* O157:H7 strain J57	*E. coli* O157:H7 strain J69
Genus	*Escherichia*	*Escherichia*	*Escherichia*
Species	*Escherichia coli*	*Escherichia coli*	*Escherichia coli*
Genome size (bp)	5,117,276	5,039,443	5,034,351
GC content (%)	50.22	50.53	50.54
Number of contigs	110	43	42
N50	128,653	222,575	336,704
L50	13	7	5
Number of systems	386	388	385
Number of RNAs	101	71	80
Number of coding sequences	5053	5016	4941
SRA ascension number	SRR25209855	SRR25082194	SRR25008992
GenBank ascension number	JAVLRS000000000	JAVCZL000000000	JAURAD000000000

**Table 2:** Virulence factors and genes detected in the three *E. coli* O157:H7 genomes.

VF class	Virulence factor	J-32	J-57	J-69
Adherence	CFA/I fimbriae	*cfaA, cfaB, cfaC*	*cfaA, cfaB, cfaC*	—
	*E. coli* common pilus (ECP)	*ecpA, ecpB, ecpC, ecpD, ecpE, ecpR*	*ecpA, ecpB, ecpC, ecpD, ecpE, ecpR*	*ecpA, ecpB, ecpC, ecpD, ecpE*
	*E. coli* laminin–binding fimbriae (ELF)	*elfA, elfC, elfC, elfG*	*elfA, elfC, elfC, elfG*	*elfA, elfC, elfC, elfG*
	*eaeA*	*eaeA*	*eaeA*	*eaeA*
	Hemorrhagic *E. coli* pilus (HCP)	*hcpA, hcpB, hcpC*	*hcpA, hcpB, hcpC*	*hcpA, hcpB, hcpC*
	Type I fimbriae	*FimA, fimC, fimD, fimE, fimF, fimG, fimH, fimI*	*FimA, fimC, fimD, fimE, fimF, fimG, fimH, fimI*	*FimA, fimC, fimD, fimE, fimF, fimG, fimH, fimI*
	Long polar fimbriae	*lpfA*	*lpfA*	*lpfA*

Autotransporter	Antigen 43	*Agn43*	*Agn43*	*Agn43*
	Cah	*Cah*	*Cah*	*Cah*
	Contact-dependent inhibition CDI system	*cdiA*	*cdiA*	*cdiA*
	EhaA	*ehaA*	*ehaA*	*ehaA*
	EhaB	*ehaB*	*ehaB*	*ehaB*
	UpaG adhesin	*upaG/ehaG*	*upaG/ehaG*	*upaG/ehaG*

Invasion	Invasion of brain endothelial cells (Ibes)	*IbeB, ibeC*	*IbeB, ibeC*	*IbeB, ibeC*
	Tia/Hek	*Tia*	*tia*	*tia*

Iron uptake	Heme uptake	*chuA*	—	*ChuA, chuS, chuT, chuU, chuW, chuX, chuY*

Non-LEE–encoded TTSS effectors		*espL1, espL4, espR1, espX1, espX4, espX5*	*espL1, espL4, espR1, espX1, espX4, espX5*	*espL1, espL4, espR1, espR3, espX1, espX2, espX4, espX5, espX6, espY1*

Secretion system	ACE T6SS	*aec15, aec16, aec17, aec18, aec19, aec22, aec23, aec24, aec25, aec26, aec27/clpV, aec28, aec30, aec31, aec32*	*aec15, aec16, aec17, aec18, aec19, aec22, aec23, aec24, aec25, aec26, aec27/clpV, aec28, aec29, aec30, aec31, aec32*	*aec15, aec16, aec17, aec18, aec19, aec22, aec23, aec24, aec25, aec26, aec27/clpV, aec28, aec29, aec30, aec31, aec32*

Toxin	Hemolysin/cytolysin A	*hlyE/clyA*	*hlyE/clyA*	*hlyE/clyA*

**Table 3:** Classes of antibiotics and their corresponding antimicrobial-resistant genes that were detected in the *E. coli* O157:H7 genomes.

Drug class	J32	J57	J69
	Resistant genes		
Aminoglycoside	*TolC, baeS, cpxA*	*TolC, baeS, cpxA*	*TolC, baeR, cpxA*
Peptide	*TolC*	*PmrF, TolC*	*PmrF, TolC*
Tetracycline	*marA, evgA, acrA, acrB, TolC, H-NS*	*marA, evgA, acrA, acrB, TolC, H-NS*	*emrK, marA, evgA, acrA, acrB, TolC, H-NS*
Macrolide	*evgA, mdtE, TolC, H-NS*	*evgA, mdtE, TolC, H-NS*	*evgA, mdtE, TolC, H-NS*
Fluoroquinolone	*marA, evgA, mdtE, acrA, acrB, TolC, AcrE, H-NS, emrR, emrB*	*marA, evgA, mdtE, acrA, acrB, TolC, AcrE, H-NS, emrR, emrB*	*marA, evgA, mdtE, acrA, acrB, TolC, AcrE, emrR, emrB, mtdH*
Penam	*marA, evgA, mdtE, acrA, acrB, TolC, AcrE, H-NS*	*marA, evgA, mdtE, acrA, acrB, TolC, AcrE, H-NS*	*marA, evgA, mdtE, acrA, acrB, TolC, AcrE, H-NS*
Carbapenem	*marA, TolC*	*marA, TolC*	*marA, TolC*
Cephalosporin	*marA, acrA, acrB, TolC, AcrE, H-NS*	*marA, acrA, acrB, TolC, H-NS*	*marA, acrA, acrB, TolC, AcrE, H-NS*
Glycylcline	*marA, acrA, acrB, TolC*	*marA, acrA, acrB, TolC*	*marA, acrA, acrB, TolC, AcrE*
Cephamycin	*marA, TolC, AcrE, H-NS*	*marA, TolC, AcrE, H-NS*	*marA, TolC, AcrE, H-NS*
Rifamycin	*marA, acrA, acrB, TolC*	*marA, acrA, acrB, TolC*	*marA, acrA, acrB, TolC, AcrE*
Phenicol	*marA, acrA, acrB, TolC*	*marA, acrA, acrB, TolC*	*marA, acrA, acrB, TolC, AcrE*
Phosphoric acid	*mdtG*	*mdtG*	*mdtG*
Penem	*marA, TolC*	*marA, TolC*	*TolC, marA*
Nucleoside	*—*	*—*	*mdtN*
Monobactam	*marA*	*marA*	*marA*
Nitroimidazole	*—*	*msbA*	*msbA*
Disinfecting agents and antiseptics	*acrA, acrB, TolC, H-NS, marA*	*marA, acrA, acrB, TolC*	*marA, acrA, acrB, TolC, AcrE, mdtN*

**Table 4:** Chromosomal mutations found in bacteria genomes that mediate antimicrobial resistance.

Genes	Function	J57	J32	J69
*ParE*	Fluoroquinolone resistance	+	−	−
*ParC*	Fluoroquinolone resistance	+	+	+
*PmrB*	Polymyxin resistance	+	+	+
*PmrA*	Polymyxin resistance	+	−	−
*16S_rrsH*	−	+	+	+
*ampC*	Beta-lactams	+	+	+
*16S_rrsB*	−	+	−	+
*16S_rrsC*	−	+	+	+
*23S*	−	+	+	+

## Data Availability

The data supporting the findings of this study are included in the article. Raw data can be requested from the corresponding author.
